# Different clinical autoimmune polyglandular syndrome phenotypes among patients with thyroid diseases

**DOI:** 10.1007/s40618-026-02850-2

**Published:** 2026-03-02

**Authors:** Elisa Gatta, Ilenia Pirola, Camilla Virili, Laura Croce, Marco Centanni, Mario Rotondi, Carlo Cappelli

**Affiliations:** 1 Department of Clinical and Experimental Sciences, SSD Endocrinologia, University of Brescia, ASST Spedali Civili of Brescia, Brescia, Italy; 2 Centro per la Diagnosi e Cura delle Neoplasie Endocrine e delle Malattie della Tiroide, University of Brescia, Brescia, Italy; 3 Department of Medico-Surgical Sciences and Biotechnologies, Sapienza University of Rome, Latina, Italy; 4 Department of Internal Medicine and Therapeutics, University of Pavia, Pavia, Italy; 5 Istituti Clinici Scientifici Maugeri IRCCS, Unit of Endocrinology and Metabolism, Laboratory for Endocrine Disruptors, Pavia, Italy; 6 Department of Clinical and Experimental Sciences, SSD Endocrinologia, University of Brescia, ASST Spedali Civili Brescia, Piazzale Spedali Civili n°1, Brescia, 25121 Italy

**Keywords:** Autoimmune polyglandular syndrome, Autoimmune thyroid disease, Hashimoto’s thyroiditis, Graves’ disease

## Abstract

**Purpose:**

To characterize and compare autoimmune comorbidity patterns and temporal dynamics in patients with Hashimoto’s thyroiditis (HT) and Graves’ disease (GD) within an autoimmune polyglandular syndrome (APS) framework.

**Methods:**

We retrospectively analysed patients with an autoimmune thyroid disease (AITD) and at least one additional autoimmune disease fulfilling APS criteria, followed at a tertiary APS referral centre between 2000 and 2025. The prevalence of associated autoimmune diseases was compared between HT and GD. Time to development of subsequent autoimmune diseases was assessed using Kaplan–Meier analysis and Cox proportional hazards models, with adjustment for sex and age.

**Results:**

A total of 1,057 patients (76% women; mean age 53.7 ± 16.4 years) were included, of whom 964 had HT and 93 GD. Type 1 diabetes was the most frequent autoimmune comorbidity, but was significantly less prevalent in GD than in HT (OR 0.48, 95% CI 0.31–0.75; FDR-adjusted *p* = 0.008). GD was selectively enriched for systemic sclerosis (OR 51.54, 95% CI 10.96–242.41; FDR-adjusted *p* < 0.001) and vitiligo (OR 3.46, 95% CI 1.82–6.55; FDR-adjusted *p* < 0.001). In patients in whom AITD was the first autoimmune manifestation (*n* = 333), GD was associated with a longer latency to additional autoimmune diseases and a lower hazard of subsequent autoimmunity compared with HT (HR 0.52, 95% CI 0.37–0.73; *p* < 0.001).

**Conclusion:**

Within APS-related AITD, HT and GD anchor distinct autoimmune phenotypes with different clustering patterns and temporal trajectories. These differences have relevant clinical implications for risk stratification and personalized surveillance of patients with autoimmune thyroid disease.

## Introduction

Autoimmune thyroid diseases (AITDs), primarily Graves’ disease (GD) and Hashimoto’s thyroiditis (HT), represent the most prevalent organ-specific autoimmune disorders (ADs) in Western countries [1–3]. The prevalence of HT-related hypothyroidism in North American cohorts ranges from 4 to 21% in females and 3–16% in males [4, 5], with autoimmune mechanisms accounting for more than 90% of non-iatrogenic hypothyroidism in iodine-sufficient regions [6]. Conversely, GD is the leading cause of hyperthyroidism in Western populations, with an estimated annual incidence of 20 per 100,000 individuals [7, 8]. AITDs’ pathogenesis is multifactorial, involving genetic susceptibility, environmental triggers, and immune dysregulation [8–10].

From an immunophenotypic standpoint, studies on HT consistently highlight a dominant Th1-skewed immune response, accompanied by enhanced Th17 activity; this inflammatory milieu is further supported by increased production of immune-activating cytokines, including IFN-γ, TNF-α, and IL-2 [11, 12], which promote progressive thyroid parenchymal damage through cytotoxic CD8⁺ T-cell and macrophage-mediated mechanisms [13]. On the other hand, evidence from both intrathyroidal and peripheral immune profiling indicates no consistent or exclusive Th1 or Th2 polarization in the different clinical stages of GD [14–16].

Increasing evidence shows that AITDs may cluster with other autoimmune conditions, configuring broader clinical entities, most notably autoimmune polyglandular syndromes (APS). APS encompasses heterogeneous combinations of endocrine and non-endocrine autoimmune diseases (AID) [17, 18], which co-occur due to a common genetic background involving multiple susceptibility loci, such as class II human leucocyte antigen (HLA), lymphoid tyrosine phosphatase (PTPN22) and cytotoxic T Lymphocyte associated antigen-4 (CTLA-4) and CD 40 [1, 13, 19]. MHC II molecules present the autoantigen through the professional antigen presenting cells (APCs), to the T-cell receptor on CD4 + T lymphocyte and represent the initiation of adaptive immune response, modulating the strength of this interaction. CTLA-4 and the protein tyrosine phosphatase-22 (PTPN22) genes are powerful inhibitors of T-cell activation, and their role has been shown in several autoimmune disorders. Altered expression of CD40, a co-stimulatory signal for B-cell proliferation may enhance the shift of the Th1/Th2 balance to Th2 dominance [20].

To date, more than 100 autoimmune diseases have been characterized, each defined by specific immunopathogenic mechanisms and a partially overlapping set of genetic susceptibility factors [13], suggesting that their co-occurrence within the same individual is not stochastic, but reflects a shared underlying immune predisposition. It has been estimated that approximately up to 1/3 of the females and 1/4 of the males with clinical, subclinical, or potential AITD are predisposed to developing another autoimmune disease [21].

Yet, the extent to which GD and HT differ in their autoimmune comorbidity spectra—and whether these differences reflect distinct immunogenetic predispositions—has not been fully elucidated. Understanding these potential differences is clinically relevant for risk stratification, screening strategies, and refinement of personalized surveillance programs.

In this context, the present study aims to characterize and compare the prevalence and clustering patterns of associated autoimmune diseases in patients with GD versus HT, to explore whether distinct autoimmune profiles correspond to specific thyroid disease phenotypes.

With the aim of better clarifying the sequence of autoimmune disease onset and the latency between manifestations, we conducted a sub-analysis of patients with APS in whom the first clinical presentation was an AITD.

## Methods

### Patients

We retrospectively reviewed the medical records of all patients with AITDs and at least one additional autoimmune disease fulfilling the criteria for APS, evaluated and followed at the APS Reference Centre outpatient clinics, ASST Spedali Civili di Brescia, between January 1, 2000, and August 31, 2025. This was a single-center observational study. The study was approved by Lombardia 6 Ethics Committee (no 5517).

### Clinical data collection

Demographic data and information on the main autoimmune comorbidities—including type 1 diabetes mellitus, Hashimoto’s thyroiditis, Graves’ disease, Addison’s disease, chronic hypoparathyroidism, premature ovarian insufficiency, celiac disease, chronic atrophic gastritis, inflammatory bowel disease, rheumatoid arthritis, systemic lupus erythematosus, systemic sclerosis, Sjögren’s syndrome, mixed connective tissue disease, vasculitis, antiphospholipid syndrome, primary biliary cholangitis, autoimmune hepatitis, alopecia areata, autoimmune urticaria, myasthenia gravis, multiple sclerosis, pernicious anaemia, immune thrombocytopenia, vitiligo, seronegative arthritis, ankylosing spondylitis, psoriasis, and bullous pemphigoid—were collected and anonymized in a dedicated electronic database.

Diagnoses were confirmed following the proper Guidelines and/or Consensus Conferences for each disorder. In particular, the diagnosis of AITDs was based on standardized biochemical and immunological criteria. HT was diagnosed in the presence of positivity for thyroid peroxidase antibodies (TPOAb) and/or thyroglobulin antibodies (TgAb); patients with isolated ultrasound features of thyroiditis in the absence of circulating thyroid autoantibodies (i.e., seronegative thyroiditis) were excluded. GD was diagnosed based on the presence of TSH receptor antibodies (TRAb) associated with supportive evidence from thyroid eye disease and/or confirmatory thyroid scintigraphy when clinically indicated. The onset of AITDs was defined as the time of documented disease presentation: for GD, the onset corresponded to the first episode of biochemically confirmed hyperthyroidism attributable to GD; for HT, the onset was defined as the date of first documented thyroid autoantibody positivity or, in cases presenting initially with hypothyroidism, the date of biochemical hypothyroidism consistent with autoimmune aetiology.

### Statistical analysis

Data are presented as mean ± SD for normally distributed variables and as counts and percentages for categorical variables. Normality was assessed using the Shapiro–Wilk test. Continuous variables were compared using the Student’s t test or the Mann-Whitney U test, as appropriate. Categorical variables were compared using the χ² test or Fisher’s exact test, where applicable. For measures of association between dichotomous variables, effect size was expressed as odds ratio (OR) with 95% confidence intervals (CI). To account for multiple comparisons, p-values were adjusted using the Benjamini–Hochberg false discovery rate (FDR) method. Time-to-event analysis was performed using Kaplan–Meier survival curves, with comparisons between groups (HT vs. GD) assessed by the log-rank test. Effect size for time-to-event differences was estimated using Cox proportional hazards regression, reported as hazard ratio (HR) with 95% CI. Models were fitted for the overall cohort and stratified by sex, and a multiplicative interaction term (AITD subtype × sex) was tested to explore effect modification. A two-sided p value ≤ 0.05 was considered statistically significant. All analyses were conducted using SPSS version 20.0 (SPSS Inc., Evanston, IL, USA) and R Studio version 4.2.2.

The results are reported in compliance with the STROBE reporting guidelines for observational studies.

## Results

A total of 1,057 patients (76% women; mean age 53.7 ± 16.4 years) with AITD in the context of APS were included in the study. Among them, 964 (91%) had a diagnosis of HT. Demographic and clinical characteristics of patients are reported in Table 1. No significant differences were observed between HT and GD in sex distribution (F/M: 727/237 vs. 78/15, *p* = 0.068), age at enrolment (53.5 ± 16.6 vs. 56.3 ± 14.9 years, *p* = 0.111), or age at AITD onset (38.4 ± 16.7 vs. 38.4 ± 15.2, *p* = 0.997).

**Table 1:** Demographic and clinical characteristics of the enrolled patients

	All (*n* = 1057)	Hashimoto’s thyroiditis (*n* = 964)	Graves’ disease (*n* = 93)	*p*
Female/Male	805/252	727/237	78/15	0.068
Age at enrolment (years)	53.7 ± 16.4	53.5 ± 16.6	56.3 ± 14.9	0.111
Age at AITD onset (years)	38.4 ± 16.6	38.4 ± 16.7	38.4 ± 15.2	0.997
AITD as first disease	333 (31.5%)	293 (30.4%)	40 (43.0%)	**0.012**
Latency between AITD and subsequent AID	2 (0–9)	2 (0–8)	8 (2-14.5)	**< 0.001**

Data are expressed as mean value ± SD, median (IQR) or absolute number (percentage). Statistically significant associations (*P* < 0.05) are highlighted. AITD: autoimmune thyroid disease; AID: autoimmune diseases

**Fig. 1:**
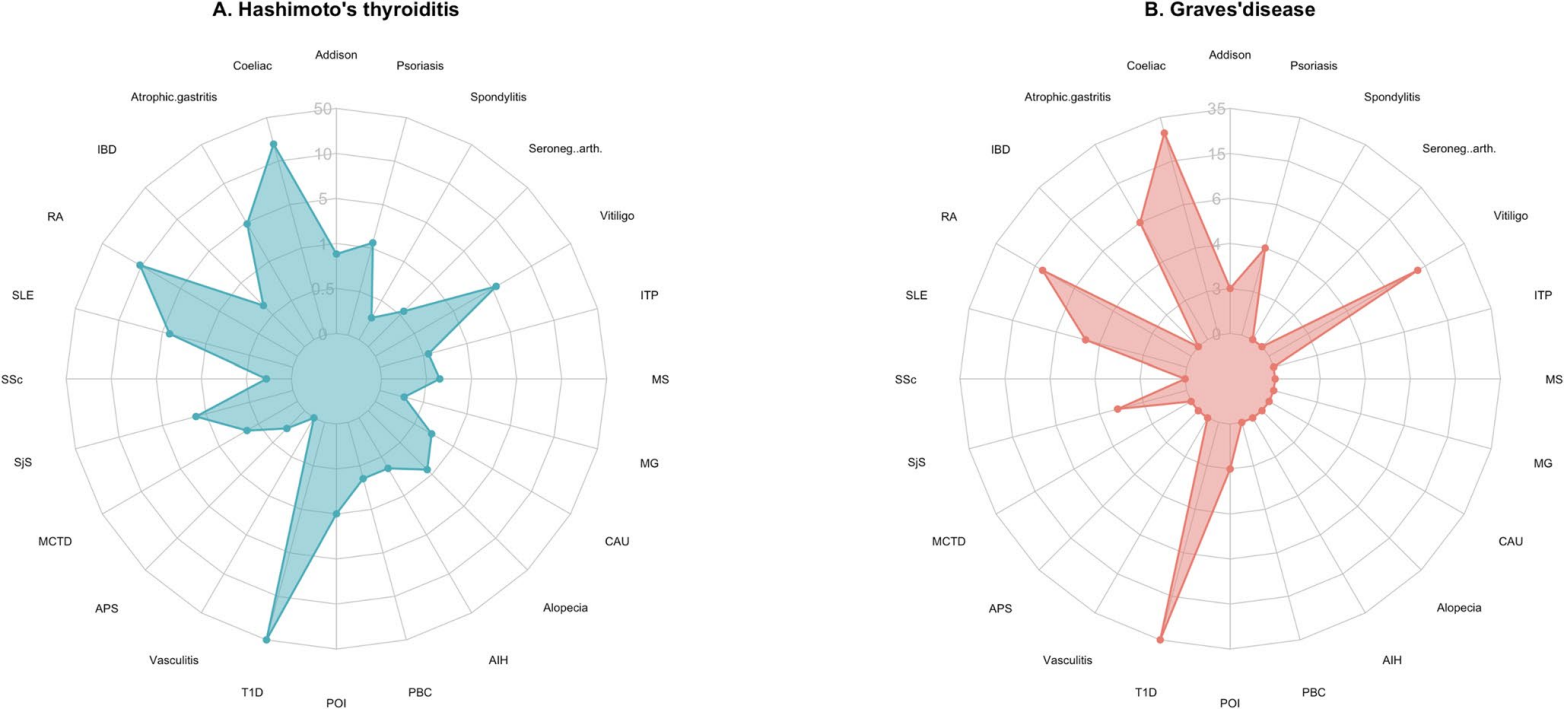
Distribution of associated autoimmune diseases in patients with (**A**) Hashimoto’s thyroiditis and (**B**) Graves’ disease. Values represent the percentage of patients with each autoimmune condition. The radial axis is plotted on a logarithmic scale. AIHA: autoimmune haemolytic anaemia; AIH: autoimmune hepatitis; APS: antiphospholipid syndrome; CAU: chronic autoimmune urticaria; IBD: inflammatory bowel disease; ITP: immune thrombocytopenia; MCTD: mixed connective tissue disease; MG: myasthenia gravis; MS: multiple sclerosis; PBC: primary biliary cholangitis; POI: premature ovarian insufficiency; RA: rheumatoid arthritis; Seroneg. arth.: seronegative arthritis; SjS: Sjögren’s syndrome; SLE: systemic lupus erythematosus; SSc: systemic sclerosis; T1D: type 1 diabetes mellitus

A total of 24 different AID was identified, allowing the classification of AITD patients as affected by APS. A two-panel radar plot illustrating the distribution of associated AID in patients who have, or will later develop, HT or GD is shown in Fig. 1. Type 1 diabetes represented the most frequent autoimmune comorbidity in both HT (52%) and GD (34%) patients. After correction for multiple testing (FDR-BH), only a subset of associations remained statistically significant. A strong differential pattern emerged for Systemic Sclerosis, which showed a markedly higher prevalence in GD (OR 51.54, 95% CI 10.96–242.41, *p* < 0.001, FDR-adjusted < 0.001). Similarly, Vitiligo was also significantly enriched in GD (OR 3.46, 95% CI 1.82–6.55, *p* < 0.001, FDR-adjusted < 0.001). Conversely, Type 1 Diabetes was significantly less frequent in GD compared with HT (OR 0.48, 95% CI 0.31–0.75, *p* < 0.001, FDR-adjusted = 0.008). A trend toward higher prevalence in GD was observed for Psoriasis (OR 3.05, 95% CI 0.98–9.46, *p* = 0.066), although this association did not remain significant after FDR adjustment (FDR-adjusted = 0.395). All other autoimmune conditions—including celiac disease, rheumatoid arthritis, Sjögren’s syndrome, and organ-specific gastroenteric and hepatic autoimmunity—showed no statistically significant differences between HT and GD after multiple testing correction (Table 2).

**Table 2:** Autoimmune comorbidity profile in patients with autoimmune thyroid diseases, comparing Graves’ disease (GD) vs. Hashimoto’s thyroiditis (HT)

Disease	OR (GD vs. HT)	CI 95% — lower	CI 95% — upper	*p* unadjusted	*p* adjusted (FDR-BH)
Addison’s Disease	2.33	0.50	10.96	0.251	0.603
Alopecia	0.49	0.03	8.36	0.999	0.999
Antiphospholipid Syndrome	5.23	0.47	58.21	0.242	0.603
Atrophic Gastritis	1.48	0.61	3.57	0.433	0.611
Autoimmune Hepatitis	2.08	0.24	18.04	0.425	0.611
Chronic Autoimmune Urticaria	0.79	0.04	14.11	0.999	0.999
Coeliac Disease	1.13	0.70	1.84	0.608	0.811
Immune Thrombocytopenia	1.14	0.61	21.37	0.999	0.999
Inflammatory Bowel Disease	2.08	0.24	18.04	0.425	0.611
Mixed Connective Tissue Disease	2.08	0.24	18.04	0.425	0.611
Multiple Sclerosis	2.08	0.24	18.04	0.425	0.611
Myasthenia Gravis	5.23	0.47	58.21	0.242	0.603
Premature Ovarian Insufficiency	2.89	0.79	10.54	0.118	0.472
Primary Biliary Cholangitis	0.93	0.05	17.01	0.999	0.999
Psoriasis	3.05	0.98	9.46	0.066	0.395
Rheumatoid Arthritis	0.95	0.52	1.72	0.857	0.999
Seronegative Arthritis	2.61	0.29	23.59	0.370	0.611
Sjögren’s Syndrome	1.98	0.56	6.91	0.230	0.603
Spondylitis	2.06	1.00	43.21	0.999	0.999
Systemic Lupus Erythematosus	1.55	0.59	4.08	0.381	0.611
Systemic Sclerosis	51.54	10.96	242.41	**< 0.001**	**< 0.001**
Type 1 Diabetes	0.48	0.31	0.75	**< 0.001**	**0.008**
Vasculitis	31.28	1.27	773.39	0.088	0.422
Vitiligo	3.46	1.82	6.55	**< 0.001**	**< 0.001**

Data are expressed as odds ratio (OR) with 95% confidence interval (CI). *p (unadjusted)* represents the nominal p-value from χ² (Pearson) or Fisher exact test, where appropriate. *p adjusted (FDR-BH)* indicates p-values corrected for multiple comparisons using the Benjamini–Hochberg false discovery rate method. Statistically significant associations (*P* < 0.05) are highlighted. GD: Graves’ disease; HT: Hashimoto’s thyroiditis; OR: odds ratio; CI: confidence interval; FDR-BH: false discovery rate (Benjamini–Hochberg)

**Fig. 2:**
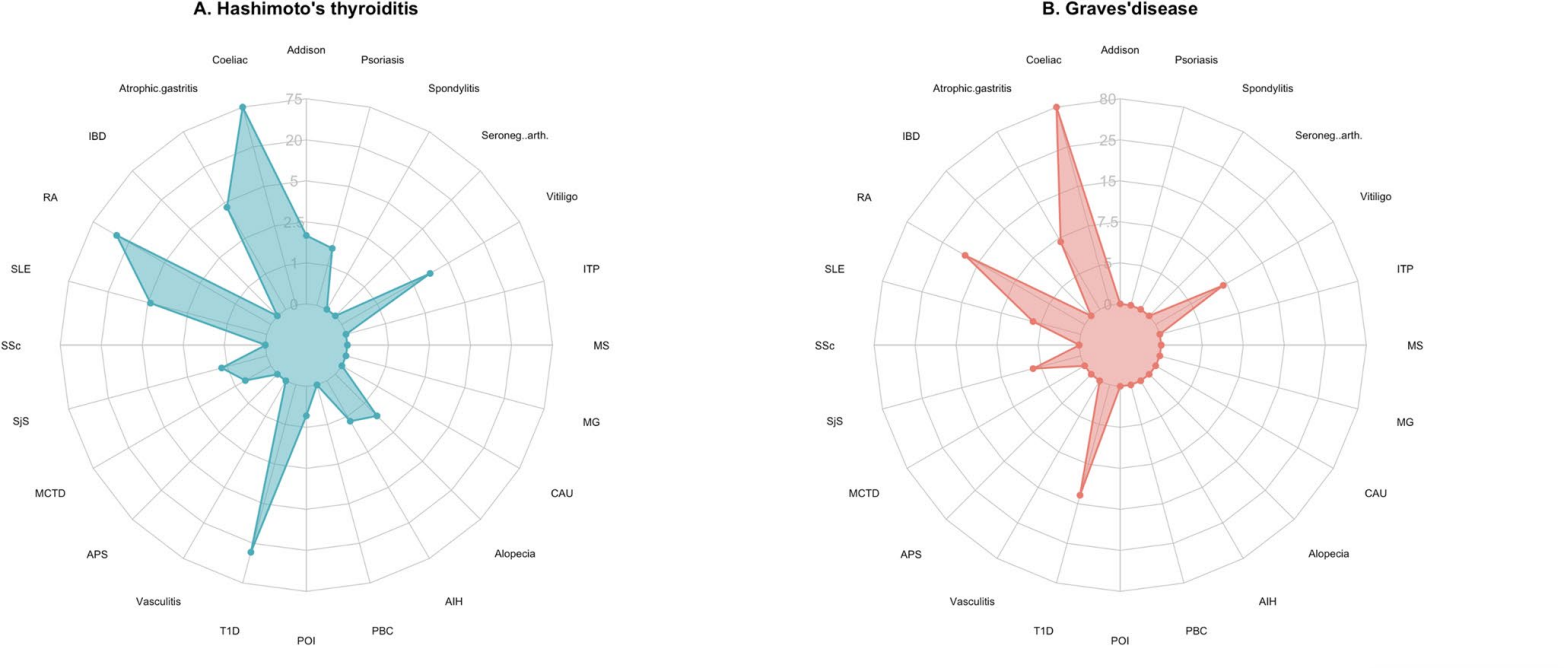
Distribution of associated autoimmune diseases in patients in whom the first autoimmune disease was (**A**) Hashimoto’s thyroiditis and (**B**) Graves’ disease. Values represent the percentage of patients with each autoimmune condition. The radial axis is plotted on a logarithmic scale. AIHA: autoimmune haemolytic anaemia; AIH: autoimmune hepatitis; APS: antiphospholipid syndrome; CAU: chronic autoimmune urticaria; IBD: inflammatory bowel disease; ITP: immune thrombocytopenia; MCTD: mixed connective tissue disease; MG: myasthenia gravis; MS: multiple sclerosis; PBC: primary biliary cholangitis; POI: premature ovarian insufficiency; RA: rheumatoid arthritis; Seroneg. arth.: seronegative arthritis; SjS: Sjögren’s syndrome; SLE: systemic lupus erythematosus; SSc: systemic sclerosis; T1D: type 1 diabetes mellitus

**Fig. 3:**
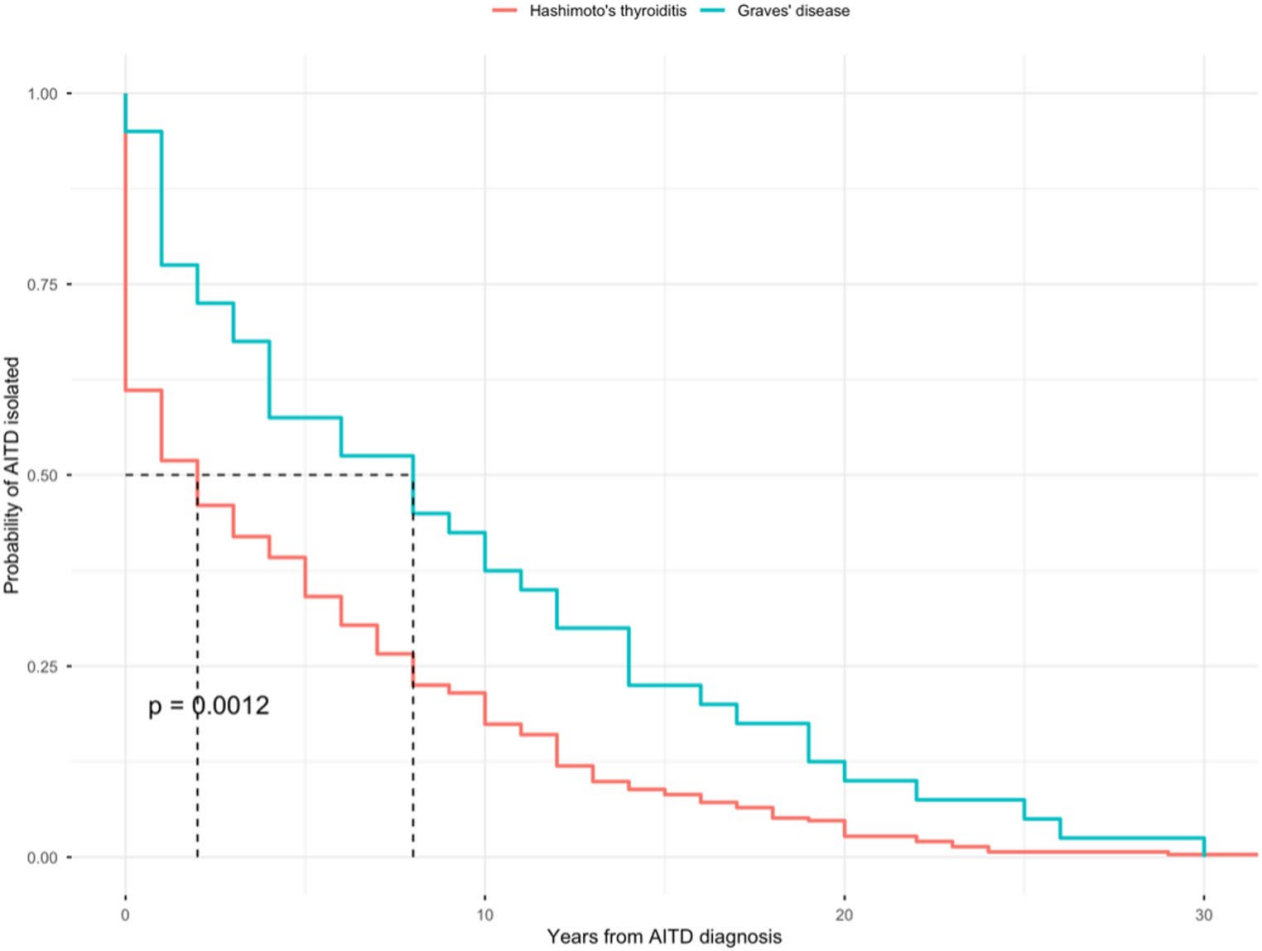
Kaplan–Meier curves showing time to development of a non-thyroid autoimmune disease in patients with autoimmune thyroid disorders

A subset analysis was performed in 333 patients (87% women; mean age 55.7 ± 13.3 years) who developed APS, in whom the first clinical manifestation was an AITD. Among them, 293 (88%) had a diagnosis of HT (Table 1). A two-panel radar plot illustrating the distribution of associated AID stratified by the first disease (HT vs. GD) is shown in Fig. 2. No significant differences were observed between HT and GD in sex distribution (F/M: 251/42 vs. 37/3, *p* = 0.236), age at enrolment (55.7 ± 15.6 vs. 55.6 ± 13.6 years, *p* = 0.267), or age at AITD onset (39.0 ± 15.7 vs. 33.5 ± 12.2, *p* = 0.080). Overall, the mean time between the diagnosis of AITD and the onset of a subsequent AID was 5.5 ± 7.4 years. A trend toward sex-related differences was noted, with longer latency in females than in males (5.8 ± 7.6 vs. 3.7 ± 5.8 years, *p* = 0.071). When females were stratified by age using 50 years as a surrogate cutoff for menopausal status, a significantly shorter latency to the development of additional AID was observed in over 50 compared with under 50 women (3.1 ± 4.2 vs. 6.5 ± 8.0 years, *p* = 0.003). Additionally, patients affected by GD showed a longer latency compared with those with HT [8 (2-14.5) vs. 2 (0–8) years, *p* < 0.001] (Fig. 3). Effect size for time-to-event differences was estimated using Cox proportional hazards regression, showing that GD was associated with a lower hazard over time and a longer latency to the onset of additional autoimmune diseases, compared with HT (HR = 0.52, 95% CI: 0.37–0.73, *p* = 0.00012). After stratification by sex, this result was confirmed in females (HR = 0.54, 95% CI: 0.38–0.77, *p* < 0.001), showing in males a similar trend not reaching statistical significance (HR = 0.40, 95% CI: 0.12–1.31, *p* = 0.13). The interaction term between sex and AITD subtype was not significant (*p* = 0.57), indicating that the effect of GD on reducing the hazard of additional autoimmunity did not differ meaningfully between sexes.

## Discussion

In this large cohort of patients with AITD within the context of APS, HT represented the predominant AITD subtype, consistent with its known higher prevalence in the general population [4, 5, 7, 8]. The demographic similarity between HT and GD groups—particularly regarding sex distribution, age at enrolment, and age at AITD onset—indicates that differences in autoimmune comorbidity patterns are unlikely to be explained by baseline characteristics alone.

HT and GD exhibit peculiar clinical and immunological profiles, despite sharing a common genetic background (e.g., HLA-DR3, HLA-DR4, CTLA4, PTPN22); indeed, they may cluster within families or, less frequently, coexist in the same individual [13]. The abovementioned differences are mirrored in the heterogeneous patterns of polyautoimmunity observed in our cohort. Type 1 diabetes was significantly less frequent in GD, consistent with the tighter association of HT with destructive organ-specific autoimmunity driven by T-cell–mediated mechanisms [13], even if this finding should be interpreted with caution given the potential influence of differential screening practices across autoimmune conditions [22]. Conversely, systemic sclerosis and vitiligo were markedly enriched in GD, suggesting shared immunogenetic features with non–organ-specific, antibody-mediated autoimmune disorders.

From an immunological perspective, HT is dominated by Th1/Th17-mediated inflammation, with secretion of IFN-γ, IL-2, TNF-α, and IL-17 and progressive parenchymal destruction driven by CD8⁺ T cells and macrophages [11–13]. GD, in contrast, reflects a less straightforward immune phenotype together with the pathogenic action exerted by TSH receptor autoantibodies, which stimulate thyroid function and thyrocyte proliferation [13, 23–25]. These divergent pathways likely contribute to the preferential clustering of HT with organ-specific autoimmune diseases and of GD with systemic antibody-mediated disorders.

Our findings are consistent with previous studies indicating that autoimmune thyroid diseases do not share a uniform pattern of autoimmune clustering. For example, in a large multicenter cohort, Boelaert et al. reported a higher prevalence of rheumatoid arthritis and pernicious anaemia in both Hashimoto’s thyroiditis and Graves’ disease patients [26]. Wiebolt et al. demonstrated a markedly greater clustering of Addison’s disease in Hashimoto’s thyroiditis and, similarly to our study, a higher prevalence of type 1 diabetes in patients with Hashimoto’s thyroiditis, supporting the concept of disease-specific autoimmune phenotypes [27]. Earlier investigations into systemic autoimmune overlap have shown that Hashimoto’s thyroiditis is more frequently associated with mixed connective tissue disease than Graves’ disease [28]. Furthermore, Ruggeri et al., focusing specifically on Hashimoto’s thyroiditis, demonstrated that age at presentation influences autoimmune clustering patterns, with systemic and rheumatologic conditions being more prevalent in adults, while type 1 diabetes and celiac disease predominate at younger ages [29]. While these studies were largely cross-sectional and based on prevalence estimates, our study extends previous observations by adopting a time-to-event approach within an autoimmune polyglandular syndrome framework, allowing the assessment of differences not only in autoimmune clustering but also in the temporal dynamics of disease development.

For other autoimmune comorbidities—including celiac disease, rheumatoid arthritis, Sjögren’s syndrome, and organ-specific gastrointestinal or hepatic autoimmunity—no significant differences were observed between HT and GD after FDR correction, although limited sample size and case dispersion across rare conditions may have reduced statistical power. Finally, HT and GD may lie on a continuous immunological spectrum influenced by environmental and immunomodulatory factors. This “immunological pendulum,” documented in a minority of patients transitioning between the two phenotypes, highlights the intrinsic plasticity of thyroid autoimmunity and complicates phenotypic classification within APS [30, 31].

The subset analysis of patients in whom AITD was the first autoimmune manifestation provides insight into the temporal dynamics of polyautoimmunity. The average latency of approximately five years between AITD onset and subsequent AID is consistent with the slow, progressive nature of autoimmune activation. Although sex differences did not reach statistical significance, the trend toward longer latency in women is compatible with the recognized role of sex hormones in modulating immune responses. Male sex confers partial protection against autoimmunity through both hormonal and Y-chromosome–linked mechanisms [32, 33]. Testosterone exerts broad immunosuppressive effects by reducing pro-inflammatory cytokine production, limiting Th1 differentiation and NK-cell cytotoxicity, and enhancing anti-inflammatory pathways [32]. When autoimmunity does occur in men, however, it may indicate a particularly susceptible immune background, consistent with the Carter effect, a genetic epidemiology concept whereby the less frequently affected sex requires a higher genetic or environmental liability to manifest disease, often resulting in more severe phenotypes or stronger familial aggregation [34]. These sex-related immunological differences may contribute to the patterns observed in our cohort, including the shorter latency to additional autoimmunity in postmenopausal women. The menopausal transition is characterized by a reduction in the immunomodulatory influence of oestrogens and a shift toward a more pro-inflammatory milieu. High oestrogen levels enhance Th2 responses and promote the expansion of regulatory T cells [35–37]; their decline after menopause may therefore facilitate Th1-mediated autoimmune activation in predisposed individuals.

Taken together, these results highlight important differences in autoimmune clustering and timing according to AITD subtype, age, and—potentially—sex. Clinically, they underscore the need for individualized monitoring strategies in APS-prone patients: more intensive surveillance may be warranted in those with HT and in postmenopausal women, whereas patients with GD may follow a slower autoimmune trajectory. These insights contribute to a more refined understanding of APS pathogenesis and may help inform future guidelines on long-term management of patients with AITD at risk for additional autoimmunity.

A methodological limitation concerns the accuracy of AITD onset dating. While GD is typically diagnosed promptly due to the rapid appearance of overt hyperthyroidism, HT may remain clinically silent for years, with a prolonged euthyroid phase preceding overt hypothyroidism. This latent period may lead to misclassification of the true onset of HT, potentially inflating the apparent latency to subsequent autoimmune manifestations. In addition, another potential limitation is the possibility of detection bias affecting the observed prevalence of type 1 diabetes among autoimmune thyroid disease subtypes. Current clinical standards recommend routine screening for thyroid autoimmunity at the time of type 1 diabetes diagnosis [22], which may increase the likelihood of identifying concomitant autoimmune thyroid disease—particularly HT—thereby partially contributing to the higher prevalence of type 1 diabetes observed in this group.

Despite these limitations, our study provides a detailed characterization of autoimmune clustering and timing in AITD-related APS. The findings highlight that HT and GD anchor distinct trajectories of systemic autoimmunity with implications for clinical surveillance. Future longitudinal studies with more precise dating of disease onset and larger samples of rare AIDs are needed to confirm these patterns and to refine risk-stratified monitoring strategies for patients with AITD at risk of polyautoimmunity.

## Data Availability

Original data generated and analysed during this study are included in this published article.
